# From induction to secretion: a complicated route for cellulase production in *Trichoderma reesei*

**DOI:** 10.1186/s40643-021-00461-8

**Published:** 2021-10-22

**Authors:** Su Yan, Yan Xu, Xiao-Wei Yu

**Affiliations:** 1 Lab of Brewing Microbiology and Applied Enzymology, School of Biotechnology, Jiangnan University, Wuxi, 214122 People’s Republic of China; 2 Key Laboratory of Industrial Biotechnology, Ministry of Education, School of Biotechnology, Jiangnan University, Wuxi, 214122 People’s Republic of China

**Keywords:** *Trichoderma reesei*, Cellulase, β-Glucosidase, Transporter, Induction, Transcription factor, Environmental factor, Secretion

## Abstract

The filamentous fungus *Trichoderma reesei* has been widely used for cellulase production that has extensive applications in green and sustainable development. Increasing costs and depletion of fossil fuels provoke the demand for hyper-cellulase production in this cellulolytic fungus. To better manipulate *T. reesei* for enhanced cellulase production and to lower the cost for large-scale fermentation, it is wise to have a comprehensive understanding of the crucial factors and complicated biological network of cellulase production that could provide new perspectives for further exploration and modification. In this review, we summarize recent progress and give an overview of the cellular process of cellulase production in *T. reesei*, including the carbon source-dependent cellulase induction, complicated transcriptional regulation network, and efficient protein assembly and trafficking. Among that, the key factors involved in cellulase production were emphasized, shedding light on potential perspectives for further engineering.

## Introduction

The cellulolytic fungus *Trichoderma reesei* is one of the most widely applied microorganisms for cellulase production. Approximate 100 g/L extracellular cellulase could be produced in *T. reesei* (Cherry and Fidantsef 2003). The secreted cellulase mainly consists of two major cellobiohydrolases CBHI/CEL7A and CBHII/CEL6A, endoglucanases EGI/CEL7B and EGII/CEL5A, and β-glucosidases BGLI/CEL3A which account for 90% extracellular protein (de Paula et al. 2018). Besides, the hemicellulase, LPMOs (lytic polysaccharide monooxygenases), CIP (cellulose-induced protein) and swollenin which belong to the secretome of *T. reesei* could also participate in the efficient degradation of biomass (Gupta et al. 2016). Such a brilliant cellulase-producing ability is increasingly attractive due to the rapid depletion of fossil fuels and the demand for sustainable development. Traditionally, the bioconversion of biomass which needs a large amount of cellulase is costly. Thus, it is critical to reducing the cost of biodegradation by improving cellulase output. *T. reesei* possesses excellent cellulase production ability, but it only harbors the minimum number of cellulase encoding genes among other fungi (Martinez et al. 2008). The specific regulators and unique cellulase response mechanism may give *T. reesei* superb cellulase production ability and greater potency in a saprotrophic habitat.

Great effort has already been made to identify the crucial factors involved in the cellulase production in *T. reesei*. Recently, numerous studies for deeper understanding and genetic engineering of cellulase production in *T. reesei* were conducted, including the identification of new regulators, characterization of the key signal transduction pathways and the discovery of the special factors that function in cellulose sensing (Chen et al. 2016; Karimi Aghcheh et al. 2014; Xu et al. 2014; Zhang et al. 2019; Zou et al. 2018). The mechanisms and critical factors in cellulase production were gradually uncovered in this genetic era.

To better engineer *T. reesei* for cellulase production, it is wise to have a comprehensive understanding of the crucial factors and complicated biological network of cellulase production that could provide new perspectives for rational modifications. Previous reviews have mainly focused either on metabolic analysis (Kubicek et al. 2009), transcriptional regulation (Druzhinina and Kubicek 2017) or secreting pathways (Saloheimo and Pakula 2012; Wang et al. 2020) for rational improvement of cellulase production. However, cellulase production in *T. reesei* is a complicated process, and the relevant pathways are simultaneously regulated through multiple factors that have not been fully elucidated. In this article, based on current studies, the cellular process of cellulase production in *T. reesei*, consisting of carbon source-dependent cellulase induction, complicated transcriptional regulation network, and efficient protein assembly and trafficking, are broadly reviewed, giving new perspectives for further exploration and engineering.

## Carbon source-dependent cellulase induction

### Cellulase production is induced by cellulose and its oligosaccharides

**Fig. 1:**
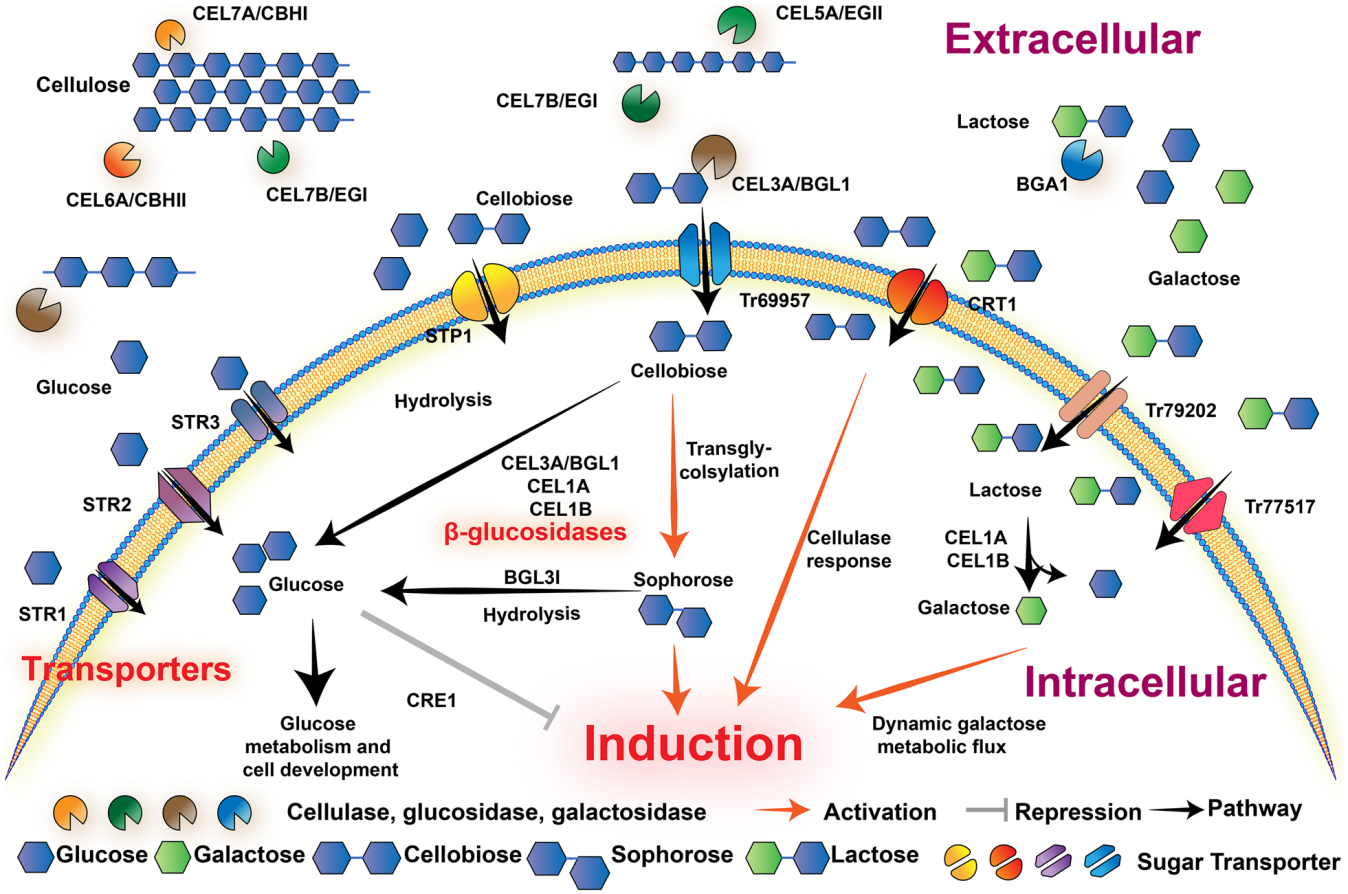
Overview of cellulase induction in *T. reesei*. This figure shows the process of cellulase induction on cellulose and lactose. Initially, the cellulase CEL7A/6A, CEL7B/5A and β-glucosidase CEL3A are involved in the degradation of cellulose, and BGA is essential for the cleavage of lactose. Next, the released mono- and disaccharides could be transported intracellularly through sugar transporters and participate in different metabolic pathways. Among that, different β-glucosidases are critical for efficient cellulase production either on cellulose or lactose, through mediating the accumulation and formation of inducer. The key components and factors are depicted in corresponding areas

Cellulase production is efficiently induced using cellulose as the carbon source in *T. reesei*. However, insoluble cellulose could never be taken in and participated in the cell metabolism. Besides, cellobiose was verified for partly cellulase induction in *T. reesei* (Mandels and Reese 1960), drawing speculation that the released cellobiose from lignocellulose induces cellulase production. Moreover, the basal transcription of CEL5B and the conidial-bound cellulases (mainly CEL6A) are also involved in induction initiation (Fig. 1), which has been reviewed by (Kubicek et al. 2009). Furthermore, the sophorose, which is transglycosylated from cellobiose by β-glucosidases, could significantly trigger cellulase induction, shedding light on the critical role of β-glucosidases in cellulase induction.

In *T. reesei*, 11 β-glucosidases were annotated which belong to the GH1 and GH3 family (Hakkinen et al. 2012), functioning in oligosaccharides hydrolysis. Besides, fungal β-glucosidase is also a transferase, which could form β-linked disaccharides with the presence of glucose and cellobiose (Sternberg and Mandels 1979). Indeed, sugar transglycosylation is important for cellulose-based cellulase induction, which could form inducer and trigger efficient inducing cascade. BGL1/CEL3A is the most abundant extracellular β-glucosidases that participate in cellulase induction. The *bgl1* null mutant showed delayed cellulase induction. However, the induction defect could be restored with the addition of sophorose, indicating the significant role of transglycosylation by β-glucosidase (Fowler and Brown 1992). However, Mach et al. (1995) showed a different result, that a *bgl1*-deleted mutant showed less cellulase induction using sophorose as the sole carbon source. Further work in which all β-glucosidase activities were blocked through the addition of β-glucosidase inhibitor nojirimycin totally impaired the cellulase induction on sophorose, suggesting that the β-glucosidase might also exert an unknown function on cellulase induction through forming another inducing component (Mach et al. 1995). These results suggest a more complex process to elucidate. Meanwhile, intracellular β-glucosidases also play a significant role in cellulase induction through balancing the hydrolysis and transglycosylation activity. The deletion of *cel1a* resulted in decreased cellulase induction using either Avicel or cellobiose as a carbon source but not using sophorose, which might be attributed to decreased intracellular transglycosylation (Shida et al. 2015; Zhou et al. 2012). Moreover, a single-nucleotide mutation of V409F influencing substrate binding in CEL1A compromised the intracellular β-glucosidase hydrolysis activity. The delayed cleavage and consumption of cellobiose, intracellularly, results in a higher accumulation of sophorose that would further increase the cellulase induction on Avicel and cellobiose (Shida et al. 2015). In addition, the appropriate amount of cellobiose was shown to be essential for efficient cellulase induction, that the cellobiose addition could rescue the induction defect on Avicel in the glucosidase null mutants (Zhou et al. 2012). Besides, β-glucosidase BGL3I was also reported to induce cellulase through mediating sophorose cleavage (Zou et al. 2018). In addition, β-glucosidase CEL3D might also be involved in cellulase induction, although the mechanism needs further investigation (Li et al. 2016). Recently, the β-glucosidases were comprehensively investigated for their cellular distribution and function in cellulase induction (Pang et al. 2021). Different β-glucosidases were shown to harbor varied localizations, which might engage in different cellular processes for cellulase induction. And the multi-copy of glucosidase resulted in comparable or decreased cellulase production compared to the parent strain (Pang et al. 2021), which might be attributed to increased disaccharides hydrolysis.

In brief, cellulase induction on cellulose in *T. reesei* is a complicated process that is coordinated by different factors, such as the saccharides concentration, specific activity of cellobiose hydrolysis and transfer, different roles of β-glucosidases in cellulase induction, etc. (Fig. 1). Significantly, the 11 different β-glucosidases could serve as critical factors in cellulase induction. Except for three well-characterized β-glucosidases, CEL3A, CEL1A and CEL1B, other β-glucosidases which show different catalytic properties (Guo et al. 2016) are also critical for efficient cellulase induction. Further study would focus on those poorly investigated glucosidases which might unravel the regulating mechanism of cellulase induction.

### Cellulase induction on lactose

In addition to cellulose and its oligosaccharide derivatives, the heterodisaccharide lactose has also been reported to induce cellulase production at a moderate level (Bischof et al. 2016). Lactose is a byproduct of the food industry and is present at very low concentrations, if at all, in the habitat of *T. reesei*, which prefers a saprotrophic lifestyle. Compared to cellulose, soluble and economically friendly lactose is more suitable for industrial cellulase fermentation (Li et al. 2017; Liu and Qu 2019). Hence, the mechanism of lactose induction seems to be preferably explored and engineered for further improvement of cellulase induction on lactose.

The utilization of lactose is initiated from the cleavage of the lactose into glucose and galactose, which is mainly conducted by extracellular β-galactosidase BGA1 or intracellular β-glucosidases CEL1A and CEL1B, which harbor β-galactosidase activities (Seiboth et al. 2005; Xu et al. 2014). Besides, two intracellular CEL1A and CEL1B are critical for cellulase induction on lactose due to the Δ*bga1* strain showed a lower growth rate on lactose but no change in cellulase induction (Seiboth et al. 2005) (Fig. 1). In contrast, the elimination of intracellular β-galactosidase activity through the deletion of *cel1a* and *cel1b* totally impaired cellulase production on lactose (Xu et al. 2014). β-Glucosidase BGL3I has also been described to increase the transcription level of cellulase genes on lactose, mainly through indirectly controlling extracellular lactose hydrolysis and lactose transport (Zou et al. 2018).

After cleavage, the released glucose serves as a repressor of cellulase induction due to CRE1-mediated CCR (carbon catabolite repression), and galactose might play a crucial role in cellulase induction (Karaffa et al. 2006). The utilization of galactose usually requires the cooperation of multiple enzymes in different metabolic pathways, including the Leloir pathway and alternative D-galactose pathway, which was illustrated by Kubicek et al. (2009). It was assumed that the metabolic rate and flux of galactose and the formation of intermediate metabolite would influence the cellulase induction. Karaffa et al. (2013) also reported a correlation between inner galacto-oligosaccharides and cellulase production, suggesting that the inducer was produced through metabolism. Moreover, the galactose addition could also rescue the cellulase induction defect in Δ*cel1a* mutant on lactose medium, although it was independent of the galactose metabolism (Seiboth et al. 2007).

The cellulase induction on lactose is an intricate process, which was well-coordinated by the intracellular glucosidases and galactose metabolic flux (Fig. 1). Similar to the pattern of cellobiose in cellulose-based induction, the hydrolysis of lactose and the accumulation of galactose both give an indispensable role in cellulase induction. Meanwhile, lactose metabolism shares a tight connection with transcriptional factors, such as XYR1 and ACE3 (Stricker et al. 2007; Zhang et al. 2019), indicating a dynamic regulation by transcription factors and metabolism networks. Furthermore, the indispensable of β-glucosidase CEL1A in cellulase induction on lactose (Xu et al. 2014) raises the possibility of other unraveled functions of glucosidase in cellulase induction, such as the formation of other potency inducers which could efficiently trigger inducing cascades. Based on the result till now, it is still elusive to draw a conclusion of the clear mechanism of cellulase induction on lactose. However, the critical factors are gradually investigated, which gives a clue for further research, and it values a lot to engineer cellulase production on lactose which would significantly lower the cost for cellulase production.

### Sugar transporters are engaged in cellulase induction

In cellulolytic fungus *T. reesei*, sugar transporters are essential for the perception of cellulose and the uptake of soluble disaccharides, which is critical for efficient cellulase induction (Nogueira et al. 2020). In *T. reesei*, only a few disaccharide transporters have been characterized. For example, the cellobiose transporter STP1 is also capable of glucose transport, and the absence of *stp1* resulted in increased cellulase induction on cellulose, which is attributed to decreased glucose input (Zhang et al. 2013). Meanwhile, another disaccharide transporter CRT1 was verified for lactose and cellobiose transport (Havukainen et al. 2020; Porciuncula et al. 2013), importantly, CRT1 is also critical for efficient signal cascade transmission that the absence of CRT1 resulted in totally impaired cellulase induction (Zhang et al. 2013). Besides, Tr79202 and Tr77517 are also capable of lactose transport, and the cellulase induction on lactose is compromised in its deletion mutant (Porciuncula et al. 2013). Moreover, the transporter Tr69957 which is involved in cellobiose, mannose and xylose transport participates in the regulation of a few cellulolytic genes in the presence of sugarcane bagasse (Nogueira et al. 2018).

Except for characterized disaccharide transporters, some monosaccharide transporters also participate in the efficient cellulase induction. For example, the xylose transporter STR1 which is capable of glucose transport was upregulated in the presence of straw (Ries et al. 2013). Besides, other characterized xylose (glucose) transporters STR2 and STR3 (Sloothaak et al. 2016) were also highly transcribed in a hyper-cellulase mutant in our study (our unpublished data). Moreover, the highly upregulated transporters in a tubulin-disrupted (Δ*tubB*) strain are also critical for increased cellulase production probably via varied cellobiose uptake (Shibata et al. 2021). And the genes related to transmembrane transport were enriched in our study (data not shown), indicating the important role of sugar transporter which still needs exploration. In addition, the induction and function of transporters are also under the subtle regulation of the endogenous regulation system. About 10 sugar transporters were shown to be probably regulated through two well-characterized transcriptional factors XYR1 and CRE1 (de Paula et al. 2018), which represent the induction pattern on cellulose and repression pattern on glucose, respectively. As shown above, the sugar transporters are suggested to be involved in the efficient cellulase induction through controlling sugar uptake flux which directly affects the accumulation of inducer for downstream inducing cascade. Moreover, the inevitable role of transporter CRT1 in cellulase induction along with other signal transduction pathways discussed in the next chapter also gives the possibility that other unraveled transporters also share such critical roles in cellulase induction, which need further investigation. Meanwhile, the regulation through transcriptional factors indicates that the inducing signal is also under the feedback regulation by inner inducing signals, the activated transcriptional factors by inducing signal might further induce the expression of transporters and control the sugar uptake. Thus, although great effort has been made in fungal sugar transporter, this field is still poorly explored, which represents a potential for genetic engineering (Nogueira et al. 2020).

## Cellulase induction is regulated at the transcription level

**Fig. 2:**
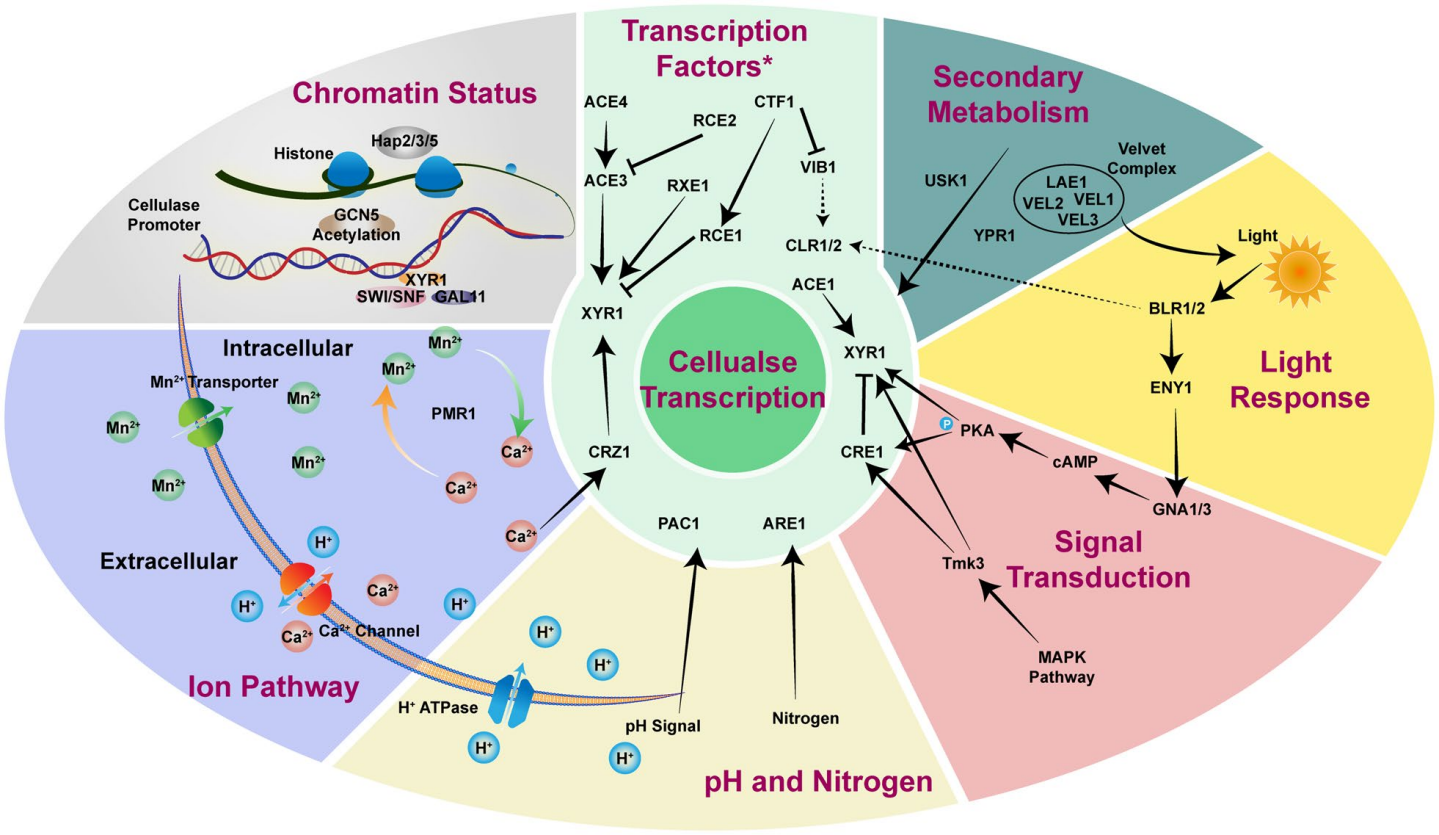
Cellulase regulation at the transcription level. In *T. reesei*, transcription of cellulase is under complicated and coordinated regulation networks including transcription factors, signal transduction, chromatin status, secondary metabolism and environmental factors such as light, nitrogen source, pH and metal ion, which are depicted in different areas with diverse colors. Only the factors involved in the crosstalk with other factors and pathways are depicted in the picture. The inter-regulation of different factors and regulators is shown by black arrows and lines. The dashed lines indicate characterized regulation mechanism in other fungi but not in *T. reesei*

Transcriptional regulation is critical for cellulase induction in *T. reesei*, which is mainly manipulated by transcription factors (TFs). Approximate 700 transcription factors were annotated in the genome of *T. reesei* (Martinez et al. 2008), while only a few of them has been investigated yet. Besides, other factors such as inner and environmental factors are also involved in transcriptional regulation, which builds a coordinated network for fine-tuned cellulase production (Fig. 2).

### Characterized transcription factors involved in cellulase induction

Cellulase genes are induced on cellulose via the transcriptional activation by the master regulator XYR1, which controls the expression of a majority of CAZymes genes (Dos Santos Castro et al. 2016). When glucose is present, cellulase induction is repressed through CRE1-mediated CCR (carbon catabolite repression), which is broadly functioned in eukaryotes (de Assis et al. 2021; Portnoy et al. 2011a). Besides, other characterized transcription factors, such as ACE2, ACE3, BglR, etc., involved in efficient cellulase production have already been investigated and reviewed previously (de Paula et al. 2018; Druzhinina and Kubicek 2017; Gupta et al. 2016), and the key information is briefly listed in Table 1.

**Table 1:** Transcription factors involved in cellulase regulation in *T. reesei*

TFs	Downstream genes	Binding sequences	Reference
Activator			
XYR1	Majority of cellulases	GGC(A/T)_3_	(Kiesenhofer et al. 2018)
ACE2	Majority of cellulases	GGCTAATAA	(Portnoy et al. 2011b)
ACE3	Majority of cellulases	CGGAN(T/A)_3_	(Zhang et al. 2019)
ACE4	*cel7a, ace3, cel6a, xyr1*	Two adjacent -GGCC-	(Chen et al. 2021b)
BglR	β-Glucosidases (except for *cel3a*)	–^a^	(Nitta et al. 2012)
CLR1/2	Xylanase, cellulase, *xyr1*, *xpp1*	–	(Beier et al. 2020a)
RXE1	*xyr1*	–	(Wang et al. 2019a)
AZF1	*cel7a, cel45a, swo1*	–	(Antonieto et al. 2019)
CLP1	*cel7a, cel7b*	–	(Wang et al. 2019b)
PAC1	Cellulase, xylanase	GCCARG^b^	(Hakkinen et al. 2015; He et al. 2014)
CRZ1	*xyr1,cel7a*	(T/G)GGCG or GGGC(G/T)	(Chen et al. 2016)
ARE1	*cel7a, cel6a, cel7b, cel5a*	HGATAR	(Qian et al. 2019)
VIB1	Cellulase	–	(Ivanova et al. 2017; Zhang et al. 2018a)
Repressor			
CRE1	Majority of cellulases, *xyr1*	SYGGRG	(Portnoy et al. 2011a)
ACE1	Majority of cellulases, *xyr1*	AGGCA	(Saloheimo et al. 2000)
RCE1	*cel7a, cel7b, cel3a*	GGC(A/T)_3_	(Cao et al. 2017)
RCE2	Majority of cellulases, *xyr1*, *cre1* and *ace3*	(T/A)NNNNCCG and CGGNNNN(T/A)	(Chen et al. 2021c)
CTF1	*vib1, rce1, ace3*	–	(Meng et al. 2020)
YPR1^c^	Cellulase and SOR clusters	–	(Derntl et al. 2016; Zhang et al. 2021)

^a^Indicates uncharacterized binding sequences

^b^Indicates components and sequences verified in other fungi, not in *T. reesei*

^c^Indicates YPR1 is a activator for SOR clusters, but its overexpression has negative effect for cellulase expression. And we classify YPR1 into the repressor for cellulase

Moreover, in the past 5 years, many new TFs were characterized, and their regulatory mechanisms were gradually uncovered (Table 1). CLR1 and CLR2 in *T. reesei* have been identified as the homologs of CLR1 and CLR2 in *Neurospora crassa* (Coradetti et al. 2012). These genes are crucial for cellulase induction in *N. crassa*, but in *T. reesei*, CLR1 and CLR2 have a minor effect on cellulase and instead are mainly responsible for xylanase regulation upstream of XYR1 and XPP1 in response to light (Beier et al. 2020a). Similarly, RXE1 was identified as an activator of XYR1 through direct binding to the *xyr1* promoter (Wang et al. 2019a), together with CRE1 modulating the cellulase induction on different carbon sources. The C_2_H_2_ zinc finger TF AZF1 in *T. reesei* has been reported to directly bind to the promoters of *cel7a*, *cel45a* and *swo1* and positively regulate their transcription on Avicel (Antonieto et al. 2019). Moreover, the plant homeo domain (PHD) protein CLP1 could also activate cellulase transcription with XYR1 at the promoter of *cel7a* and *cel7b* (Wang et al. 2019b), although the precise mechanism needs further investigation. Recently, it was found that a cellulase regulator RCE1 specifically served as a repressor for cellulase induction through directly bound to the *cel7a* promoter, competing with XYR1 for the same recognition site (Cao et al. 2017). In addition, a repressor CTF1 was isolated via an artificial zinc finger library, and the deletion of *ctf1* caused a global increase in cellulase gene expression (Meng et al. 2020). Another activator ACE4 was also shown to participate in the cellulase induction mainly through regulating the expression of ACE3 (Chen et al. 2021b). Recently, the biological function of a novel repressor RCE2 was characterized to repress expression of a majority of cellulase genes, and transcription factors such as *cre1*, *xyr1* and *ace3*. A binding competition between RCE2 and ACE3 was also observed, indicating a synergetic regulation among RCE2 and other transcription factors (Chen et al. 2021c).

### Other factors involved in transcriptional regulation

Aside from these specific transcription factors, other factors involved in secondary metabolism, signal transduction and chromatin status regulation also participate in the transcriptional modification of cellulase production in different aspects (Fig. 2).

#### Secondary metabolism

The velvet protein complex, which consists of LaeA, VeA, VelB and VelC in *Aspergillus nidulans*, is conserved in fungi and regulates secondary metabolism and photo-dependent development (Bayram and Braus 2012). The homolog of LaeA in *T. reesei* is annotated as LAE1 and controls the activation of XYR1 on cellulase induction. The transcription of *xyr1* was impaired in the *lae1* null mutant, and the constitutively expressed XYR1 could not rescue the induction defect in Δ*lae1*, indicating that LAE1 is an upstream regulator of XYR1 (Seiboth et al. 2012). VEL1 is the homolog of VeA in *A. nidulans* and shows a function similar to that of LAE1 upon cellulase induction in *T. reesei* (Karimi Aghcheh et al. 2014). Further function investigation through *vel1*, *vel2* or *vel3* deletion also verified their critical role in secondary metabolism due to reduced sporulation, as well as the diminished cellulase expression especially in Δ*vel1* and Δ*vel2*, indicating a more significant role of VEL1 and VEL2 in this regulon (Liu et al. 2016). These results all demonstrate the significant role of the velvet protein complex in cellulase induction as well as secondary metabolism regulation. Besides, the overexpression of the transcription factor YPR1, which is involved in polyketide synthase (PKS)-encoding genes regulation, dramatically increased sorbicillin production, but the cellulase production was decreased mainly through unbalanced regulation between cellulase production and secondary metabolism (Zhang et al. 2021). And the protein kinase USK1 in the vicinity of sorbicillin producing genes could also regulate secondary metabolism and cellulase production in darkness (Beier et al. 2020b).

#### Signal transduction

Transcription factor VIB1 in *T. reesei* was also investigated because of a distal chromosome translocation in a cellulase-negative mutant QM9978, which causes a loss of function of VIB1 (Ivanova et al. 2017). The overexpression of VIB1 enhanced cellulase production but with lower biomass accumulation (Zhang et al. 2018a). VIB1 in *N. crassa* controls many pathways, including carbon signal transduction and CCR, and it could also regulate cellulase expression through modulating CLR2 (Zhang et al. 2018a); thus, in *T. reesei,* the function of VIB1 and how VIB1 could regulate cellulase production needs further investigation. The mitogen-activated protein kinase (MAPK) pathway, which is ubiquitous in eukaryotes and is involved in biological regulation, could also participate in the regulation of cellulase expression through the phosphorylation of transcription factors by TMK3 in *T. reesei* (Wang et al. 2013). Although the deletion of another transcription factor TMK1 in MAPK pathway could increase cellulase production, it is independent of the transcriptional regulation of cellulase expression (Wang et al. 2017). Moreover, the cellulase inducing signal received through heterotrimeric G proteins receptors activates the Gα subunit, mostly GNA1/3 (responsible for cellulase induction)(Schmoll et al. 2009; Seibel et al. 2009), and elevates downstream cAMP abundance. The main target of cAMP in this cascade is protein kinase A (PKA), which could phosphorylates other transcription factors, such as the main repressor CRE1 in CCR (Cziferszky et al. 2002), and other upstream factors regulating *xyr1* expression (Schuster et al. 2012). The CRE1 phosphorylation is crucial for its repression function that a E244V mutation which leads to dephosphorylation and loss of DNA binding ability (Cziferszky et al. 2002). A subtle exchange of C-terminal phosphorylation site S388 to valine resulted in an unphosphorylated state, which improved cellulase production regardless of CCR in the presence of glucose (Han et al. 2020a, b). This data collectively suggested an inevitable function of phosphorylation of normal regulation of transcription factor, which serves as promising candidate for engineering.

#### Chromatin status

In eukaryotes, DNA is wrapped around histones to form the nucleosome; the transcription and activation of genes sometimes require the release of histones and trigger nucleosome repositioning. The HAP2/3/5 complex, which is widely conserved in eukaryotes, could also participate in chromatin remodeling. The transcriptional activation of *cbh2/cel6a* in *T. reesei* has been reported as the result of the depletion of nucleosome occupancy downstream of CAE (*cbh2*-activating element) under induction conditions (Zeilinger et al. 2003). The negative regulator CRE1 was also noted for its significant role in nucleosome positioning under repression conditions, combined with the upregulation of SWI/SNF complex (Ries et al. 2014). Further work on XYR1 also demonstrated that XYR1 is indispensable for chromatin remodeling and transcription activation under cellulose induction through binding to TrSNF12 and TrGAL11 at its acidic activating domain (Cao et al. 2019; Zheng et al. 2020a). Moreover, transcription activation also requires GCN5-mediated histone acetylation, which affects chromatin status (Xin et al. 2013).

### Transcriptional regulation via environmental factors

It is essential for microorganisms to sense environmental factors, such as light, nitrogen, pH and metal ions, to maintain homeostasis and biorhythms. Cellulase production in *T. reesei* is also under the regulation of several environmental factors at the transcription level (Fig. 2).

#### Light

Light is a significant signal that can regulate cell metabolism and rhythm. In *T. reesei*, ENY1 has been reported as a regulatory protein that influences cellulase expression in response to light (Schmoll et al. 2005). Further study has identified two photoreceptors, BLR-1 and BLR-2, the homologs of Wc-1 and Wc-2 in *N. crassa*, which can sense light and activate the transcription of *eny1* (Castellanos et al. 2010). Then, the downstream G-protein α subunits GNA-1 and GNA-3 are activated and affect the downstream cAMP and PKA (protein kinase) pathway, indicating crosstalk between light sensing and signal transduction (Schmoll et al. 2009; Seibel et al. 2009; Tisch et al. 2014). Additionally, the study of cAMP in response to light showed that deletion of adenylate cyclase *acy1* and cAMP-dependent protein kinase A *pkac1* disturbs the cellulase regulation in response to light (Schuster et al. 2012), indicating the significance of light regulation for cellulase expression via the cAMP pathway. Besides, the phospholipase C *plc-e* was verified for its vital role in inducing signal cascade between cAMP and downstream Ca^2+^ signaling pathway (Chen et al. 2021a), which is depicted in detail below. Besides, the light response cellulase production was also regulated via velvet protein complex, that the decreased cellulase expression was only shown in dark, but almost unchanged in light condition in Δ*vel3 T. reesei* (Liu et al. 2016). Meanwhile, the light receptor white-collar complex (WCC) has also been reported to directly bind to the promoter of *clr-1* in *N. crassa* (Smith et al. 2010), which suggests a direct regulation by light. And a positive regulation by ENY1 on major cellulase activator such as XYR1 and ACE3 also give a indispensable role of light regulation among transcription factors (Schmoll 2018).

#### Nitrogen sources

In addition, different nitrogen sources could also influence cellulase production. ARE1 is responsible for protease production through direct regulation of *apw1* and *apw2* in *T. reesei*. Further study confirmed that it could also regulate the transcription of *cel7a* and *cel6a* when cultured with (NH_4_)_2_SO_4_ as nitrogen source (Qian et al. 2019), which demonstrates crosstalk between cellulase production and nitrogen metabolism.

#### pH

Environmental pH can influence the basal metabolism of fungi (Li et al. 2013), and it can also affect cellulase production. A transcription factor PAC1 in *T. reesei* has been characterized as a homolog of PacC in *A. nidulans*, and it can receive pH signals through the *pal* pathway and regulate cellulase production in neutral pH (He et al. 2014). Although several cellulase genes are pH-responsive, relatively few cellulase genes are under PAC1 regulation (Hakkinen et al. 2015), suggesting the presence of an alternative regulator that could influence cellulase transcription with different pH signals. Moreover, PacC in *A. nidulans* was shown to directly bind to the GGCARG motif for its regulatory activity (Espeso et al. 1997), while the detailed function of PAC1 in *T. reesei* has not yet been investigated. In addition, the balance of intracellular and extracellular pH is maintained by H^+^-ATPase (Liu et al. 2019b). The acidic metabolites produced during cell development would lower the intracellular pH, thus, proton exchange via different pumps is indispensable for pH homeostasis. In *T. reesei*, the H^+^-ATPase Tr76238 could efficiently output H^+^. Deletion of the H^+^-ATPase would increase the intracellular H^+^ concentration and stimulate Ca^2+^ input through Ca^2+^ channels, which then would increase cellulase transcription through the Ca^2+^ signal transduction pathway (Chen et al. 2016).

#### Metal ions

The Ca^2+^ signal transduction pathway in eukaryotes has been widely reported to be involved in diverse biological processes, including cell development and environmental stress responses (Hagiwara et al. 2008). Mandels and Reese first reported that the addition of 2.5 mM Ca^2+^ increased cellulase production in *T. reesei* (Mandels and Reese, 1957), and Chen et al. uncovered the calcineurin-dependent Ca^2+^ signal transduction pathway and identified the regulator CRZ1 (calcineurin-responsive zinc finger transcription factor 1) (Chen et al. 2016), which is involved in regulating *xyr1* and *cel7a* transcription. The addition of Mn^2+^ was also suggested to increase cellulase production in *T. reesei* by triggering the Ca^2+^ signal pathway (Chen et al. 2018). Two Mn^2+^ transporters, TPHO84‑1 and TPHO84‑2, were also identified, whose deletion showed lower cellulase production compared to that with Mn^2+^ addition. Besides, the Ca^2+^/Mn^2+^ homeostasis is maintained through the Ca^2+^/Mn^2+^ transporter PMR1, the deletion of which became less sensitive to extracellular Mn^2+^ addition (Chen et al. 2018). The reason for this might be explained by the disordering of Ca^2+^/Mn^2+^ balance and the blocking of CRZ1-mediated transcriptional activation. The Ca^2+^ signal transduction pathway seems to be the dominant ion transduction pathway in *T. reesei* that could engage in crosstalk with other pathways and influence cellulase production (Fig. 2). Although the Ca^2+^ signal transduction pathway and its interaction with the Mn^2+^ pathway were also observed in *A. nidulans* (Hernández‐Ortiz and Espeso 2013), the function of the regulator CrzA has been reported in response to cellular stress and Ca^2+^ homeostasis in *A. nidulans*, but not yet for others. Recently, the traditionally used solvent N,N-dimethylformamide (DMF) has been reported for enhancing cellulase production through changing cell plasma membrane permeability (Chen et al. 2019), which then creates an intracellular Ca^2+^ burst and activates downstream Ca^2+^ signal transduction. However, other organic solvents, such as DMSO, did not have a similar effect. In *T. reesei,* the interactive work between Mn^2+^ and Ca^2+^ signal pathway, signifying the importance of Ca^2+^ in the cellulase induction, provides a new perspective on whether other metal ions might also be involved in the signaling pathway, which in turn had a positive effect on cellulase induction.

As shown above, cellulase induction is a well-coordinated process and could be influenced by different factors which finally affect cellulase induction at the transcription level via multiple transcription factors. The discovery of novel transcription factors and clarification their mechanism mean a lot for rational engineering (Sun et al. 2020; Yan et al. 2021) and partly give a clue for understanding the regulation network upon cellulase induction. However, the characterized transcription factors only account for a small percentage of all the annotated transcription factors in *T. reesei*, which is still a promising area for further investigation.

## The brilliant secreting ability for cellulase production

**Fig. 3:**
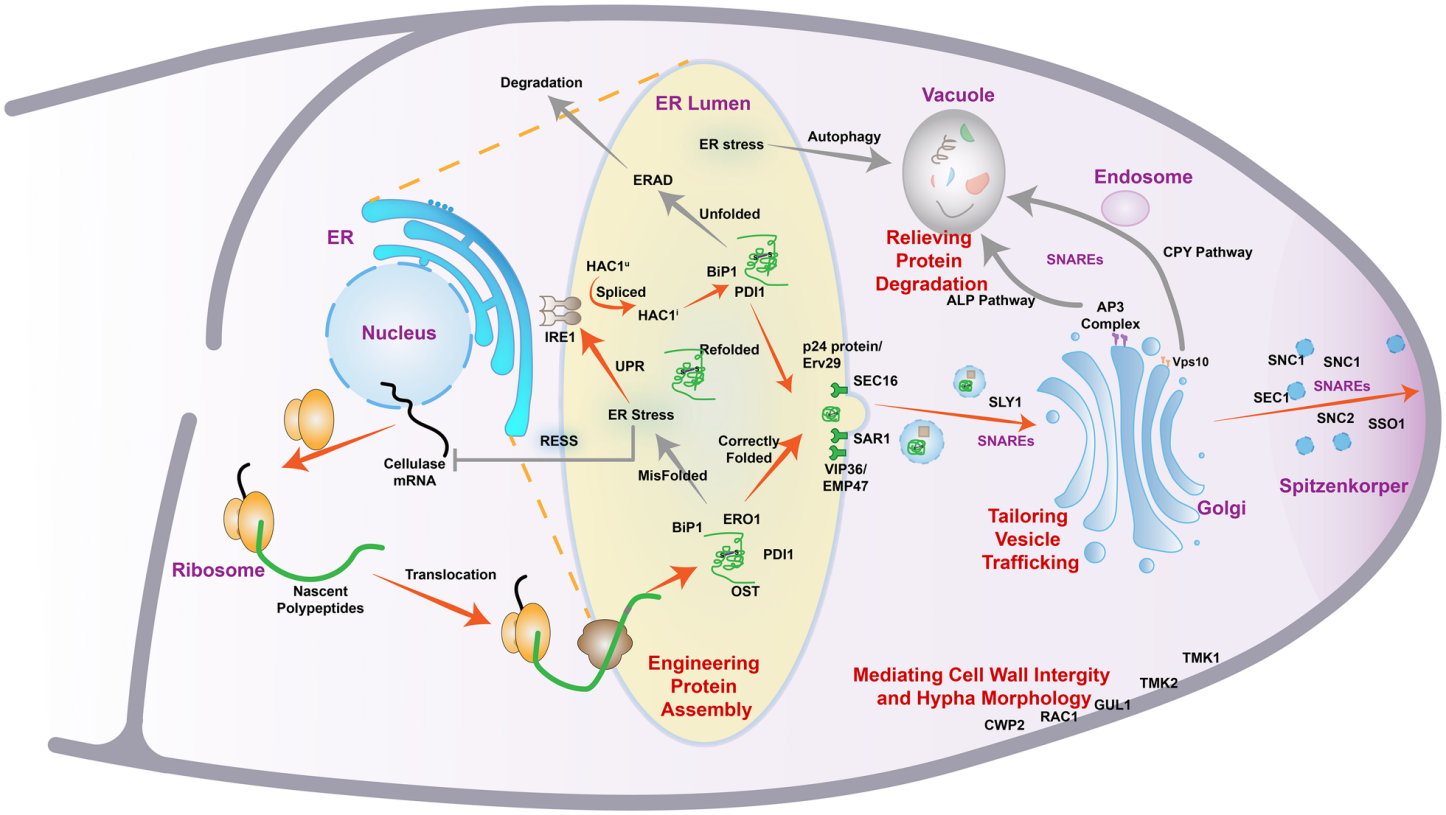
Secretion pathway in *T. reesei*. Secreted cellulase will enter the classical secretion pathway, which passes through the ribosome, endoplasmic reticulum, Golgi, and secrets extracellularly. The ER lumen is amplified to give a comprehensive review of the protein assembly and ER quality control. The basic pathway for cellulase secretin is annotated as red arrow, with the assistance of multiple factors, including the PDI1, BiP1, HAC1, SNC1, etc. Insufficient folding or degradation of the secreted protein is indicated by gray arrows. Moreover, the promising secretion factors for improved cellulase production in *T. reesei* are also annotated aside, which need further exploration. The cellular structures are marked by purple, and the promising secretion pathways for engineering are shown with red font

Same as other eukaryote microorganisms, *T. reesei* can also systematically process secreted protein through folding and proper decoration. As the desired platform for cellulase production, exceed 100 g/L cellulase could be secreted in *Trichoderma* species (Cherry and Fidantsef 2003), drawing strong focus on how could this species secretes such a huge amount of cellulase. Traditionally, the protein secretion underwent the classical secretion pathway including translation in the ribosome, folding in the endoplasmic reticulum (ER), glycosylation in Golgi and finally transported extracellularly. A detailed review of this classical secretion pathway in yeast and other fungi has already been investigated and could be found elsewhere (Celinska and Nicaud 2019; Shoji et al. 2008). In this section, the characterized secretion factors in *T. reesei* involved in efficient cellulase production are reviewed. Meanwhile, the critical pathways characterized in other fungi are also emphasized, which shed light on further investigation in *T. reesei* (Fig. 3).

### Efficient protein assembly and quality control in ER

The cellulase secretion is initiated from the protein translation in the ribosome, then followed by the intricate assembly in the endoplasmic reticulum (ER). The polypeptides entering into the ER lumen need to be folded with the assistance of binding protein BiP1 and protein disulfide isomerase PDI1. BiP1 is the homolog of Kar2 in *S. cerevisiae*, which belongs to the HSP 70 family, functioning in the folding of proteins in the ER. PDI1 is involved in the formation of disulfide bonds with pdi1 oxidase ERO1 (Conesa et al. 2001). Moreover, the preliminary glycosylation is also conducted in ER via the oligosaccharyltransferase OST and ER-resident proteins (Sun and Su, 2019). The glycosylation of CEL7A in *T. reesei* considerably influences its stability and secretion (Qi et al. 2014), implying the importance of glycosylation for secreted protein in ER. The correct assembly is critical for efficient cellulase production, thus, enhancing assembly through overexpression of BiP1 could result in higher cellulase output in *T. reesei* (Gao et al. 2018). However, only comparative cellulase production was achieved in a mutant with PDI1 overexpression in *T. reesei* (Gao et al. 2018), which might be attributed to the diverse functions of its three orthologs which coordinately work to ensure the correct folding of secreted proteins (Martinez et al. 2008).

Unfortunately, the protein folding in ER does not always follow settled procedures. Incorrect and insufficient folding would trigger its ER quality control mechanism and cause the retention of unfolded protein, which increases ER stress. The ER quality control is mainly maintained by unfolded protein response (UPR) which is activated through the IRE1-mediated pathway in *T. reesei*. The detection of incorrectly folded protein could trigger downstream UPR cascade through membrane-bound IRE1, which is then coupled and autophosphorylated to form an activated IRE1 (Mori 2015). Then, the activated IRE1 could act on the maturation of the mRNA of transcription factor HAC1 through splicing (Markku et al. 2003), and then induce the expression of the downstream genes for protein folding, such as *pdi1*, *bip1*, etc. Enhanced production of foreign glucose oxidase and cellulase were achieved in *T. reesei* mutants with overexpressed *hac1*, respectively (Gao et al. 2018; Wu et al. 2017), while another study in *Pichia pastoris* gave a negative result (Guerfal et al. 2010). In addition, the expression of the activated form of *hac1* in *Aspergillus niger* is also detrimental for its growth, although the genes involved in secretion are highly upregulated (Carvalho et al. 2012). Meanwhile, the activation of UPR could also trigger endoplasmic reticulum-associated degradation (ERAD) for the degradation of unfolded proteins (Bernasconi and Molinari 2011). The knockdown of ERAD-related genes has a positive effect on protein secretion in *A. niger*, although the growth defect is also observed in its Δ*doa* mutant (Carvalho et al. 2011). In *T. reesei* Rut-C30, a frameshift of the glucosidase II alpha subunit gene (*gls2a*) functioning in glucose residue removal of the early stage of ERAD might also account for the hypersecreting phenotype (Peterson and Nevalainen 2012). Moreover, an unraveled mechanism, called repression under secretion stress (RESS), is also exclusively presented in filamentous fungi for ER stress response through downregulating the transcription of major secreted proteins, such as cellulase and glucoamylase, in *T. reesei* (Pakula et al. 2003) and A. niger (Al-Sheikh et al. 2004), respectively. The clear mechanism for RESS has not been characterized until now, but some speculation has arisen that the RESS effect seems to function independently of the UPR (Fan et al. 2015). Moreover, it might also participate in the transcriptional regulation that a cis-element ‘TCACGGGC’ motif in the *amyB* promoter is required for RESS in *A. oryzae* (Zhou et al. 2015). Besides, the deletion of adaptor protein 3 involved in alkaline phosphatase transport is also shown for decreased RESS effect, which further increases cellulase production in *N. crassa *(Pei et al. 2015). In addition, vacuolar/lysosomal degradative autophagy is also responsible for alleviating ER stress by directly delivering misfolded protein to the vacuole in A. oryzae (Kimura et al. 2011). Although the homologs of autophagy-related genes could also be found in *T. reesei*, no related reports have been published since then.

As shown above, the efficient protein assembly and the quality control mechanism in ER are critical for protein secretion. Although only a few research have been conducted in *T. reesei*, the characterized mechanism in other fungi could also give a clue for further engineering in *T. reesei*. Moreover, the effect for increased protein assembly also seemed to be related to the inner secreting stress, that the multiple-copy of secreting protein gives better results in ERAD defective mutants than that of single-copy in A. niger (Carvalho et al. 2011). Great potential remains through manipulating protein assembly, while the exact effect and strategies still need experimental exploration.

### Efficient vesicle trafficking contributes to protein hypersecretion

After intricate assembly and decoration, the folded proteins are ready for vesicle transporting which is initiated from the vesicle budding. The recognition and budding of secreting proteins are mediated by specific receptors. In *N. crassa*, p24 family proteins and Erv29p are indispensable for the delivery of the major cellulases CBHI and CBHII, respectively (Starr et al. 2018). Besides, increased α-amylase secretion was achieved in *S. cerevisiae* through SEC16 overexpression, which functions in assisting budding vesicle formation (Bao et al. 2017). Meanwhile, secreted proteins can be retained by lectin-like cargo receptors AoVip36 and AoEmp47 in *A. oryzae*, and deletion of these receptors greatly increases the protein secretion rate by alleviating the abundance of ER-residual secreting protein (Hoang et al. 2015). Although the secretion-related protein SAR1 is also involved in bud formation, sar1 overexpression did not result in improved cellulase production in *T. reesei* (Gao et al. 2018).

Moreover, the vesicle trafficking from ER to the Golgi and then from Golgi to the plasma membrane is assisted via multiple SNAREs (soluble NSF [N-ethylmaleimide-sensitive factor] attachment receptor proteins) (Shoji et al. 2008). Great effort has been made to increase protein secretion through improving vesicle trafficking. For example, increased glucose oxidase secretion has been achieved with overexpressed v-SNARE protein SNC1 in *T. reesei* (Wu et al. 2017), and the overexpression of secretion factors SSO1, SNC2, etc., is benefit for recombinant cellulase secretion in *S. cerevisiae* (Tang et al. 2017). Moreover, overexpression of Sec1/Munc18 (SM) proteins SEC1 and SLY1 facilitating vesicle trafficking increases heterologous protein production in *S. cerevisiae* (Hou et al. 2012). Although positive results have been achieved in different fungi with varied secreting proteins, the result through manipulating vesicle trafficking still seems to be protein-dependent (Tang et al. 2017), and it might not work on some occasions (Gao et al. 2018). Despite the diverse results through manipulating vesicle trafficking, it remains an ideal strategy for improving cellulase production in *T. reesei* which needs further verification.

### The biodegradation of secreting proteins in vacuole

Occasionally, the secreted proteins were shown to be visualized in the vacuole in some cases probably due to disordered trafficking system and endoplasmic reticulum stress, which results in decreased protein production (Kimura et al. 2011; Li et al. 2019; Pang et al. 2021). The vacuole is an acidic organelle that contains multiple proteases mainly functioning in protein degradation to maintain the balance of inner environmental stability. Improved protein secretion has been achieved in a Δ*vps10* mutant in *A. oryzae* through attenuating Vps10-dependent CPY pathway (Yoon et al. 2010). Similarly, our experiment also showed higher cellulase production in Δ*vps10 T. reesei* compared to its parent strain (our unpublished data). Meanwhile, alleviated vacuole transport through disrupting the μ subunit of the adaptor protein 3 (AP-3) that attenuating ALP pathway could also increase cellulase production in *N. crassa* (Pei et al. 2015). Furthermore, in *A. oryzae*, the chymosin production increased up to threefold in autophagy gene disrupted mutants compared to that in parent strain, however, the growth and conidiation defect were also observed due to disordered degradative system (Yoon et al. 2013). Although positive results have been achieved in other fungi to lower vacuole transport for improved protein secretion, several secretion factors involved in the vacuole transport might also take part in the efficient endosomal trafficking and normal cell development (Lemmon and Traub 2000; Pei et al. 2015). Thus, it is attractive to improve cellulase production through balancing the normal cell growth and vacuole degradation in *T. reesei*, although few related research has been conducted since then.

### The hypha morphology and cell wall integrity affect protein secretion

Traditionally, the cellulase secretion in *T. reesei* is a tip-directed process, and the secreted proteins are highly accumulated in the tip area which is annotated as Spitzenkorper (Saloheimo and Pakula 2012). Increased cellulase production has been achieved in mRNA-binding protein *gul1 null* mutant which increases hypha branching in *T. reesei* (Zhao et al. 2021). And the deletion of small Rho GTPase *rac1* in *T. reesei* provokes hyperbranching and results in increased cellulase production on lactose (Fitz et al. 2019). Meanwhile, the transcriptome data from *Δgul1* in *N. crassa* show the downregulation of cell wall-related genes, indicating the significance of cell wall integrity in cellulase secretion (Lin et al. 2018). The deletion of *tmk1* and *tmk2* in *T. reesei* involved in MAPK pathway both shows downregulation of β-1,3-glucan synthase and higher cellulase production, while the transcription of cellulase is almost unchanged (Wang et al. 2014, 2017). In *S. cerevisiae*, the deletion of the cell wall-encoding gene *cwp2* thins the cell wall, resulting in higher CBHI secretion (Li et al. 2020). The higher production of cellulase might be attributed to a loosened cell wall thus resulting in increased secretion efficiency.

## Conclusion and further perspectives

The rational engineering of filamentous fungus *T. reesei* for efficient cellulase production meets the demand for economical production and higher productivity in the reality of sustainable development. Great efforts have been made to better understand the molecular mechanism behind cellulase production. And the critical steps involved in cellulase production in *T. reesei* have been broadly discussed in this review, including (1) the indispensable role of β-glucosidases and sugar transporters which control the intake and formation of inducer on cellulase induction; (2) the characterized regulators that participated in the coordinated regulation of cellulase production at the transcription level; (3) and the promising secretion pathway for engineering. The comprehensive review of the critical steps in cellulase production might give new perspectives for further exploration and engineering.

Although the characterized regulators have been successfully applied for enhancing cellulase production in *T. reesei* (Gao et al. 2018; Zheng et al. 2020b; Zou et al. 2018), further study still needs to focus on the elucidation and investigation of biological function of annotated regulators which are the promising candidates for enhanced cellulase production in engineered *T. reesei*. Moreover, the design of new synthetic circuits could be a benefit for cellulase production (Zhang et al. 2018b), which gives the possibility that the design of synthetic inducing pathways tightly controlling the inducer uptake and accumulation. In addition, the development of the dynamic circuits via the utilization of conditional promoters, for example, the copper-dependent promoter *Ptcu* in *T. reesei* (Lv et al. 2015), thiamine-derepressed promoter *Pthi* in *A. oryzae* (Yoon et al. 2013) as well as the exploration of stress-induced or growth-coupled promoters could give a dynamic regulation on cellulase induction and transcriptional regulation, which might mimic the real inducing cascade in *T. reesei*. Then, the investigation of potential candidates for enhanced cellulase production still needs efficient screening via transcriptome exploration and genomic search (Liu et al. 2019a), as well as rapid genetic manipulation through novel molecular tools such as the CRISPR/Cas9 (Liu et al. 2015). Besides, the secretion pathway which is also critical for efficient cellulase production was hardly explored in *T. reesei*, and this area was far from full investigation even in filamentous fungi, which gives a clue for further exploration. However, the dynamic endosomal system in secretion pathway is related to cell growth and morphology, thus it is important to engineer cellulase secretion in a more flexible and dynamic strategy. In the future, we expect a deeper understanding and diversified methods to improve cellulase production and meet the demand for cellulase utilization in *T. reesei*.

## Data Availability

All data generated or analyzed during this study are included in this published article, or upon request from the corresponding author.

## Abbreviations

CCR: Carbon catabolite repression
TF: Transcription factor
PHD: Plant homeo domain
MAPK: Mitogen-activated protein kinase
WCC: White-collar complex
CRZ1: Calcineurin-responsive zinc finger transcription factor 1
DMF: N,N-Dimethylformamide
ER: Endoplasmic reticulum
UPR: Unfolded protein response
ERAD: Endoplasmic reticulum-associated degradation
RESS: Repression under secretion stress
